# UPF1/circRPPH1/ATF3 feedback loop promotes the malignant phenotype and stemness of GSCs

**DOI:** 10.1038/s41419-022-05102-2

**Published:** 2022-07-23

**Authors:** Jinkun Xu, Guoqing Zhang, Jinpeng Hu, Hao Li, Junshuang Zhao, Shengliang Zong, Zhengting Guo, Yang Jiang, Zhitao Jing

**Affiliations:** 1 Department of Neurosurgery, The First Hospital of China Medical University, Shenyang, China; 2 Department of Neurosurgery, Shanghai Tenth People’s Hospital, Tongji University School of Medicine, Shanghai, China

**Keywords:** Cancer stem cells, Oncogenes

## Abstract

Glioblastoma multiforme (GBM) is the most lethal type of craniocerebral gliomas. Glioma stem cells (GSCs) are fundamental reasons for the malignancy and recurrence of GBM. Revealing the critical mechanism within GSCs’ self-renewal ability is essential. Our study found a novel circular RNA (circRPPH1) that was up-regulated in GSCs and correlated with poor survival. The effect of circRPPH1 on the malignant phenotype and self-renewal of GSCs was detected in vitro and in vivo. Mechanistically, UPF1 can bind to circRPPH1 and maintain its stability. Therefore, more existing circRPPH1 can interact with transcription factor ATF3 to further transcribe UPF1 and Nestin expression. It formed a feedback loop to keep a stable stream for stemness biomarker Nestin to strengthen tumorigenesis of GSCs continually. Besides, ATF3 can activate the TGF-β signaling to drive GSCs for tumorigenesis. Knocking down the expression of circRPPH1 significantly inhibited the proliferation and clonogenicity of GSCs both in vitro and in vivo. The overexpression of circRPPH1 enhanced the self-renewal of GSCs. Our findings suggest that UPF1/circRPPH1/ATF3 maintains the potential self-renewal of GSCs through interacting with RNA-binding protein and activating the TGF-β signal pathway. Breaking the feedback loop against self-renewing GSCs may represent a novel therapeutic target in GBM treatment.

## Introduction

Glioblastoma multiforme (GBM) ranks as the most lethal primary carcinoma in the central nervous system. Patients cannot reach a better prognosis despite undergoing precise surgical resection following radiotherapy and chemotherapy [1]. The median survival time of GBM patients is often statistically less than 14.6 months. Despite the availability of chemotherapeutic agents such as Temozolomide, there is often a slight improvement in prognosis due to high recurrence [2]. Heterogeneous cancer cells underlie GBMs’ response to different treatments [3]. GSCs (glioma stem cells) rank top proliferation hierarchy in the GBM system, causing invasion and recurrence [4, 5]. Modern oncology research tells us that various tumor treatment strategies are based on targeting the intrinsic mechanism of cancer cells, as well as the relevant immune checkpoints and molecular targets, etc [6, 7]. Therefore, it is very promising to explore the mechanisms that potentially lead to the malignancy of GBM and reveal its originating principles.

Circular RNAs (circRNAs) belong to non-coding RNAs structured as unique single-stranded loops lacking 5’caps and 3’tails [8]. Their structure brings them better stability than linear RNAs and allows them to stay against RNase R digesting [9]. Such property is used for circRNAs enrichment to understand the expression of circRNAs in tissues and diseases, especially cancer [10]. Many cancer research has shown that circRNAs can function as miRNA sponges, thereby reducing miRNA-mRNA targeting in a regulatory way [11]. An increasing number of reports have identified that circRNAs could bind to proteins. Such binding would produce molecular regulatory effects distinct from the circRNAs and proteins themselves [12]. Increasing evidence revealed that circRNAs could form dynamic RNA-protein binding, which showed spatial and temporal expression profiles [13]. CircRNAs, in this way, led to tumor cells’ proliferation and invasion and elucidated the mechanism of regulatory nuclear translocation when circRNAs interacted with proteins [14, 15]. However, little has been reported about the mechanisms of circRNA-protein interaction in GBM and GSCs.

Upstream frameshift 1 (UPF1) is an RNA helicase known as the regulator of nonsense-mediated mRNA decay (NMD). It contains a premature termination codon (PTC) to protect cells from detrimental protein production [16, 17]. However, if there has a dysfunction of UPF1, it could contribute to tumor initiation [18]. It suggested that UPF1 plays an equally important role in developing tumors [19]. UPF1 can prevent cancer stem cell (CSC)-like properties and inhibit the epithelial-mesenchymal transition (EMT) process via decreasing ABCC2 expression in hepatocellular carcinoma (HCC) [20]. UPF1 also participated in tumorigenesis and cancer progression of colorectal cancer (CRC) by UPF1-mediated mRNA destabilization regulating NR4A1 [21]. In glioma, UPF1 was reported to be downregulated in U87 and LN229 cell lines after acting with LncRNA PVT1. However, relevant studies on glioma stem cells are still unavailable, and the related mechanisms still need to be explored in depth [22].

Activating transcription factor 3 (ATF3) is a transcription factor related to the proliferation and metastasis of cancer cells [23]. It is a member of the ATF/cyclic adenosine monophosphate response element-binding protein (CREB) subfamily, which got a unique leucine zipper (bZIP) domain structure that can interact with proteins in response to oncogene stimulation [24]. Research has reported its role in pathological processes such as gene transcription, maintenance of body homeostasis, cell signaling, cell death, tumor angiogenesis, and tumor invasion [25–27]. It was revealed that ATF3 expression could be regulated by circ_0001742, which acted as competing endogenous RNA through targeting miR-431-5p in tongue squamous cell carcinoma development [28]. ATF3 was reported overexpressed in glioma cohorts, and ATF3-knockdown was able to reduce the proliferative and invasive activity of glioma cell lines U373MG both in vitro and in vivo [29]. In addition, ATF3 can play a synergistic anti-tumor role with Epigenetic Drugs and Protein Disulfide Isomerase Inhibitors in tumor therapy [30].

Our study discovered that circRPPH1 (has_circ_0000512) could help GSCs become more malignant by facilitating their proliferation, invasion, and self-renewal. Then, UPF1 was identified as an RNA-binding protein that bound to circRPPH1 by pull-down assays. It maintained the stability of circRPPH1 and thus facilitated the malignancy of GSCs. CircRPPH1 was also found bound to ATF3, a transcription factor that upregulated UPF1 and Nestin expression. Therefore, a positive feedback loop was established. Simultaneously, we identified stemness markers and discovered that they were impacted by circRPPH1 in this process of expression alterations. Finally, we found that the entire regulatory mechanism improved GBM by activating the TGF-β signaling pathway. As a result of the study, we discovered a novel mechanism that explained the increased aggressiveness of GBM.

## Materials and methods

### Glioma samples and ethical approval

All glioma tissues were obtained from fresh clinical resections at the Department of Neurosurgery from January 2010 to December 2015, the first hospital of China Medical University. The total tissues are graded as 20 WHO II, 25 WHO III, 25 WHO IV, controlled by 10 adjacent brain tissues. Clinical information about molecular subtypes of glioma, such as IDH status, 1p/19q status, H3F3A status, and MGMT status for these samples, are available in Table 1. Individual consent was obtained from parents, and the procedures were under the protocol approved by the First Hospital of China Medical University research ethics committee (Approval Code: AF-SOP-07-1.1-01).

**Table 1:** Relationship of circRPPH1 expression to clinical features of glioma patients.

Clinical features		Samples (*n* = 70)	CircRPPH1 expression*		*P-value*
			Low (*n* = 35)	High (*n* = 35)	
Sex	Male	37	20	17	*P* = 0.473
	Female	33	15	18	
Age	≤50	30	18	12	*P* = 0.147
	>50	40	17	23	
WHO grade	II	20	13	7	***P*** = **0.012**
	III	24	15	9	
	IV	26	7	19	
IDH status	Wild	31	11	20	***P*** = **0.030**
	Mutant	39	24	15	
1p/19q status	Codeletion	37	23	14	***P*** = **0.031**
	Non-codeletion	33	12	21	
H3F3A status	Wild	43	26	17	***P*** = **0.027**
	Mutant	27	9	18	
MGMT status	Methylation	33	22	11	***P*** = **0.008**
	Unmethylation	37	13	24	

*CircRPPH1 expression was detected by RT-qPCR and ranked from low to high. The high expression of circRPPH1 was defined as the expression level higher than the median expression level of circRPPH1. Bold values identify statistical significance (*P* < 0.05).

### Glioma stem cells isolation and culture

Our glioma stem cells (WHO IV: GSC35–GSC40) were isolated from our fresh clinical GBM tissues. GBM tissues obtained from surgical resection were immediately immersed in the medium and transported to the laboratory on ice for further processing. The tissues were washed and minced mechanically. The tissues were then enzymatically digested into single cells using 0.25% trypsin (Gibco). The single cells were filtered via a 200-mesh cell strainer and centrifuged (400 g) for 5 min. After treating the cells with red blood cell lysis (Solarbio, Beijing, China), they were centrifuged again. Finally, the obtained cells were cultured in a serum-free medium containing DMEM/F12 (Gibco) supplemented with B27 (2%, Gibco), Recombinant human basic fibroblast growth factor (rh-bFGF, 20 ng/ml, Gibco), and epidermal growth factor (rhEGF, 20 ng/ml, Gibco) at 37 °C with 5% CO_2_. Growth factors (bFGF and EGF) were added twice a week. The stemness of GSCs was verified by neurospheres formation, multidirectional differentiation, and immunofluorescence staining. The detailed clinicopathological information is presented in Supplementary Table 1. Human GBM cell lines, U87 and LN229 cells, were purchased from American Type Culture Collection (ATCC, Manassas, VA, USA). They were firstly cultured in Dulbecco’s modified Eagle’s medium (DMEM, Gibco) with 10% fetal bovine serum (Gibco). When cells were grown in the logarithmic phase, they were enzymatically dissociated into single cells using 0.25% trypsin (Gibco). After being washed with phosphate-buffered saline (PBS, Gibco), they were cultured in the DMEM/F12 (Gibco) medium supplemented with 2% B27 (Gibco), Recombinant human basic fibroblast growth factor (rh-bFGF, 20 ng/ml, Gibco), and epidermal growth factor (rhEGF, 20 ng/ml, Gibco) at 37 °C with 5% CO_2_. Growth factors were added twice a week and the serum-free medium was replaced every 4–6 days. All of the cell lines and GSCs studied had been cultured for less than 20 generations and had passed mycoplasma and the short tandem repeat (STR) DNA profiling tests.

### Lentiviral vector construction and transfection

The lentivirus assays were performed as previously described [31]. The lentivirus-based vectors for circRPPH1 overexpression, UPF1 overexpression, ATF3 overexpression, RNAi-mediated knockdown of circRPPH1, UPF1 and ATF3, and their negative controls were all constructed by Gene-Chem (Shanghai, China). The sequences of all siRNAs are listed in Supplementary Table 2. Transfection efficacy was detected by qRT-PCR and western blotting.

### Quantitative real-time polymerase chain reaction (qRT-PCR) and northern blot

RNA of tissues and cells was obtained through a Trizol reagent extracting kit (Invitrogen, California, USA). A Prime Script RT Master Mix reagent kit (TaKaRa, Kyoto, Japan) was used to transcribe circRPPH1 and mRNA into cDNA reversely. SYBR Green Master Mix (TaKaRa) with PCR LightCycler480 (Roche Diagnostics, Basel, Switzerland) was used for the assays. Furthermore, RNase R (Epicentre Technologies, Madison, WI, USA) was treated to eliminate the effect of linear RNA on RPPH1. 2.5 mg of total RNA with 5 U/μg RNase R was incubated at 37 °C for 20 min. The RNA quality of each sample was assessed on Ultra-micro Nucleic Acid Analyzer (BioDrop Duo, biochrom, Cambridge, UK). Glyceraldehyde 3-phosphate dehydrogenase (GAPDH) was used as endogenous control and the relative expression levels were calculated using the 2^−ΔΔCT^ method. Primers used in this study are listed in Supplementary Table 3. Northern blot was performed to detect the circRPPH1 using 1.2% agarose gel where 20 μg extracted total RNA was analyzed. Photographs were taken using Image Lab software (Bio-Rad, California, USA). Actinomycin D (17559, MedChemExpress, New Jersey, USA) was used to detect the stability of the RNA. We treated GSCs with actinomycin D in a final concentration of 5 µg/mL for 0, 6, 12, 18, 24, 30 h, and then extracted the RNA for RT-qPCR detection.

### Fluorescence in situ hybridization (FISH)

To detect the location of circRPPH1 in GSCs, we designed oligonucleotide probes containing fluorescent labels complementary to circRPPH1 junction sequences by the Clone Manager suite of analysis tools. A total of 1 × 10^5^ cells were cultured on a confocal dish overnight at 37 °C with 5% CO_2_. Then RNA FISH assay was performed using a specific circRPPH1 FISH kit (BersinbioTM circRNA FISH, Guangzhou, China) according to the manufacturer’s protocols. 4,6-diamidino-2-phenylindole (DAPI, Solarbio) was used to stain cells nuclei. Photographs were taken on the confocal microscope (Olympus, Tokyo, Japan).

### Western blotting and drugs treatment

GSCs’ protein was extracted in the RIPA lysis buffer with 100 mM PMSF (Beyotime, Shanghai, China). The nuclear and cytoplasmic protein of GSCs were extracted using an extraction kit (Nuclear and Cytoplasmic Protein Extraction Kit, Beyotime). Then added protein lysates equally into wells of SDS-PAGE gels and transferred protein on a PVDF membrane after electrophoresis. The membranes were incubated with a primary antibody at 4 °C overnight and hybridized with a secondary antibody the next day for 1 h. The primary antibodies information: anti-SOX2 (1:2000, ab92494, Abcam, Cambridge, UK), anti-OCT4 (1:2000, ab200834, Abcam), anti-Nestin (1:2000, ab105389, Abcam), anti-CD133 (1:2000, Cat No: 18470-1-AP, proteintech, Wuhan, China), anti-Nanog (1:2000, ab109250, Abcam), anti-GAPDH (1:2000, ab8245, Abcam), anti-β-actin(1:2000, ab8227, Abcam), anti-UPF1 (1:10000, ab109363, Abcam), anti-ATF3 (1:1000, ab254268, Abcam). Drugs used for the assays include cycloheximide (CHX) (14126, Cayman chemical, Michigan, USA) and proteasome inhibitor MG132 (474787, Merck, Darmstadt, Germany). GSCs were treated with 10 μM CHX to block translation. MG132 was used at 50 μM for each group. For signal pathway study, cells were exposed to 5 ng/ml TGF-β1 (HY-P70543, MedChemExpress) or 5 μg/ml LY2109761 (700874-71-1, MedChemExpress). At last, membrane stripes were detected in the ECL image system (Tanon, Shanghai, China).

### Transwell invasion assay

24-well plates were treated with 600 μl DMEM containing 20% FBS. Then seed GSCs in Matrigel-coated transwell supports (Transwell3422, Corning, New York, USA) within DMEM containing 10% FBS at a total volume of 200 μl. Insert the supports into the plates and culture at 37 °C with 5% CO_2_ for 20 h. Stain the invasive cells with 0.1% Crystal Violet Solution (Beyotime), take photographs using an Inverted Microscope (Olympus), and count cell numbers using ImageJ software.

### 5-ethynyl-2′-deoxyuridine (EdU) assay

Seed GSCs in 24-well plates at 1 × 10^5^ cells per well for 20 h. Then use the EdU Cell Proliferation Kit (Beyotime) following the manufacturer’s protocol. In brief, GSCs were treated with 10 mM EdU for two hours and detected with Azide Alexa Fluor 555. Photograph the EdU positive cells using Fluorescent Inverted Microscope (Olympus) and count their percentage using ImageJ software.

### Neurospheres formation assays and extreme limiting dilution analysis

GSCs were seeded into U-bottom Ultra Low Adherence 96-well plates (Corning) at a density of 10^3^ cells per well and cultured in fresh serum-free DMEM/F12 medium with 2% B27, 20 ng/ml rh-bFGF, and 20 ng/ml rhEGF(Gibco) for 72 h. Then, the relative neurosphere size was observed through a light microscope (Olympus). For in vitro limiting dilution assay, GSCs were seeded into 96-well plates at a gradient of 1, 10, 20, 30, 40, and 50 cells per well, and each gradient was replicated 10 times. The number of neurospheres formed in each well was obtained after 7 days of incubation, and the neurosphere formation efficiency was calculated via a protocol called the Extreme Limiting Dilution Analysis (http://bioinf.wehi.edu.au/software/elda) [32].

### Neurospheres differentiation

The neurospheres which formed after two weeks of culture were transferred to centrifuge tubes, centrifuged, resuspended in Dulbecco’s Modified Eagle’s Medium (Gibco) supplemented with 10% fetal bovine serum (Gibco) and transferred to new culture flasks. After 5 days, differentiation of glioma stem cells into glioma cells from the center of the neurospheres in all directions could be observed via a light microscope (Olympus). The multi-lineage differentiation ability of GSCs was confirmed by immunofluorescence staining of GFAP (Cat No: 16825-1-AP, proteintech) and β-III tubulin (ab18207, Abcam).

### Chromatin immunoprecipitation (ChIP) assays

Chromatin immunoprecipitation was achieved by ChIP Assay Kit (Beyotime) following the manufacturer’s instructions. First, 1 × 10^6^ cells were lysed in SDS Lysis Buffer with 1 mM PMSF and ultrasonication. Then centrifuge the samples and mix the supernatant with ChIP Dilution Buffer. With an anti-ATF3 antibody or normal rabbit IgG, keep a 20 μl sample as input and add Protein A + G Agarose/Salmon Sperm DNA into the others to get an immunoprecipitated chromatin complex. Finally, use qPCR to detect the purified DNA samples. All the primers are listed in Supplementary Table 3.

### RNA immunoprecipitation assay

The RIP assays were performed through the EZ-Magna RIP RNA-binding Protein Immunoprecipitation Kit (Millipore) according to the manufacturer’s protocols. GSCs under different experimental groups were lysed in RIP buffer, including magnetic beads conjugated with negative control IgG, anti-UPF1, or anti-ATF3 antibodies (Millipore). The immunoprecipitated RNAs were isolated after incubation with proteinase K, and the precipitants were detected via qRT-PCR assays.

### RNA pull-down assay

According to the official protocols, the interaction between circRPPH1, UPF1, and ATF3 was detected using The Pierce Magnetic RNA Protein pull-down Kit (Thermo Fisher Scientific). Briefly, purified RNA was labeled with biotinylated RNA probes. Then, the positive control (Input), negative control (Antisense RNA), and biotinylated RNA were mixed and coincubated with our GSCs proteins at room temperature. Mix the RNA-protein complex with magnetic beads to prepare a probe-magnetic bead complex. Finally, the complexes were detected by western blotting after being washed and boiled, the whole process using β-actin as a control.

### Immunofluorescence

Immunofluorescence staining was performed as previously described [33]. Briefly, the GSCs were fixed with 4% paraformaldehyde (solarbio) for 15 min, permeabilized with 0.5% Triton X-100 (solarbio) for 20 min, blocked with 2% BSA (solarbio) for 1 h, and probed with primary antibodies as below: anti-ATF3 (ab254268, Abcam); anti-CD133 (Cat No: 18470-1-AP, proteintech) and anti-Nestin (ab105389, Abcam); anti-GFAP (Cat No: 16825-1-AP, proteintech) and anti-β-III tubulin (ab18207, Abcam) at 4 °C overnight. Then, all the samples were treated with fluorescein isothiocyanate or rhodamine-conjugated secondary antibodies. Subsequently, the GSCs were counterstained with DAPI (solarbio) for 5 min. Finally, the staining was visualized by a laser scanning confocal microscope (Olympus).

### Enzyme-linked immunosorbent assay (ELISA)

The ELISA was performed as previously described [31]. We used a commercial kit (Cusabio, Stratech, UK) to detect the concentration of TGF-β1 in the supernatant of the GSCs medium according to the manufacturer’s protocols. All results were normalized to the protein concentration in the control group.

### Luciferase reporter assay

Luciferase reporter assays were performed as described previously [31]. Firstly, the luciferase reporter plasmids (UPF1-wt and UPF1-mt, Nestin-wt and Nestin -mt were constructed by Gene-Chem (Shanghai, China). The GSCs were seeded into 96-well plates at a density of 5 × 10^3^ cells per well. Then they were transfected with luciferase reporter plasmids and performed other relative treatments for 48 h. Finally, we detected the relative luciferase activities using a Dual-Luciferase Reporter Assay System (Promega, USA). Relative luciferase activity was calculated as the ratio of firefly luciferase activity to Renilla luciferase activity.

### Immunohistochemistry (IHC)

IHC was performed as described previously [31]. Briefly, all samples were fixed in 10% neutral formalin and embedded in paraffin. Then, we cut them into 4 μm thick sections and used anti-Ki67, UPF1, ATF3, and TGF-β1 (Abcam) primary antibody labeling to make a combination. All sections were imaged using an optical microscope (Olympus). German immunohistochemical score (GIS) was applied to evaluate the protein staining intensity [34].

### Intracranial xenografts

For constructing a model in vivo, 6-week-old female BALB/c nude mice (Beijing Vital River Laboratory Animal Technology, Beijing, China) were raised for xenograft experiments in the Laboratory Animal Center of China Medical University, which was ethically provided by the Animal Care Committee of China Medical University (CMU2021586). The GBM model was established via stereotaxic injection in the basal ganglia region (2 mm lateral and 2 mm anterior to the bregma with a depth of 3 mm) of BALB/c nude mice (*n* = 5, per group). Each group was observed daily for distress or death signs. The overall survival times of mice were measured by Kaplan-Meier survival analysis. The brain tissues of mice were taken out immediately within 12 hours of death. Volume information of tumors was measured using the following formula: V = (D × d^2^)/2, of which D and d represented the longest and the shortest diameters of the xenograft tumor.

### Bioinformatics analysis

GEO database (GSE146463) was used to identify our candidate circRNA [35]. Starbase (http://starbase.sysu.edu.cn) and Cancer-Specific CircRNA Database (CSCD, http://gb.whu.edu.cn/CSCD/) databases were used to predict potential RNA-binding proteins interacted with circRPPH1. CatRAPID algorithm (http://service.tartaglialab.com/) was used to estimate the binding propensity of protein-RNA pairs. JASPAR database (http://jaspar.genereg.net) of transcription factor binding profiles was used to search for protein binding motifs. Patients data were obtained from the Chinese Glioma Genome Atlas (CGGA, http://www.cgga.org.cn) and the Cancer Genome Atlas (TCGA, http://cancergenome.nih.gov). And then, Gene set enrichment analysis (GSEA, http://www.broadinstitute.org/gsea/index.jsp) was used to analyze the enrichment of the signaling pathway with high versus low ATF3 expressions.

### Statistical analysis

Results are reported as mean ± SD of at least three independent experiments. All analysis for statistics was conducted using GraphPad Prism 7 for Windows or the R software. The comparisons of two independent groups were detected by the chi-square test and two-tailed Student’s t-test. The statistical significance among different groups was calculated under one-way ANOVA. For in vivo experiments, Student’s two-tailed unpaired t-test was used to determine the statistical significance of different groups. Kaplan-Meier analysis and log-rank test were used to tell the survival difference. A two-tailed P less than 0.05 was considered significant for all the statistical tests.

## Results

### CircRPPH1 is up-regulated in glioblastoma and correlated with the progression and poor prognosis

**Fig. 1:**
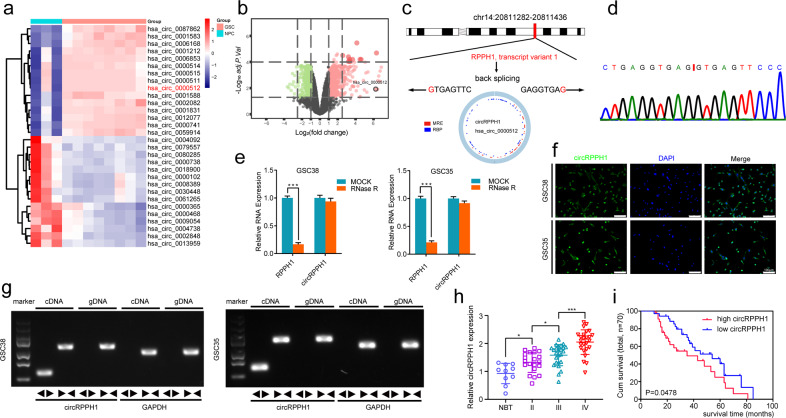
CircRPPH1 is highly expressed in glioblastoma compared with normal brain tissues. **a** Heatmap showed that circRPPH1 was highly expressed in glioblastoma in GSE146463. **b** The volcano plot demonstrated that circRPPH1 was highly expressed in the glioblastoma. **c** The schematic illustration showed the formation of circRPPH1 via back splicing. **d** Sanger sequence confirmed the head-to-tail splicing of circRPPH1. **e** Relative RNA expression between RPPH1 and circRPPH1 in GSC38 and GSC35 under RNase R digesting was tested by qPCR (*p* < 0.0001, Student’s *t*-test). **f** Fluorescence in situ hybridization (FISH) assays showed the position of circRPPH1 using a specific circRPPH1 probe. Scale bar = 100 μm. **g** Northern blotting showed the products of qPCR assays in cDNA and gDNA. **h** CircRPPH1 expressed at a higher level in WHO high grades of glioma than in normal brain tissues, tested by qPCR (grade II, *n* = 20; grade III, *n* = 25; grade IV, *n* = 25; NBT, *n* = 10, II vs. NBT. *p* = 0.0114; III vs. II. *p* = 0.0232; IV vs. III. *p* = 0.0002; One-Way ANOVA). **i** Kaplan-Meier analysis showed high circRPPH1 expressed patients suffered a poor prognosis than the low expressed ones. (For each group, *n* = 35, *p* = 0.0478, Log-rank test). All results are obtained as the mean ± SD under at least biological triplicate assays. *p < 0.05, **p < 0.01, ***p < 0.001.

We first performed circRNA data analysis in GSE146463 between normal and tumor tissues. The results showed the differentially expressed circRNAs with fold change >1.0 and *p* < 0.05 (Fig. 1a, b). We selected the top ten of these highly expressed circRNAs in GBM. We then examined their actual expression using our GBM samples and we found that circRPPH1 was the most highly expressed among the ten candidates. So finally we selected circRPPH1 for further investigation. CircRPPH1 (has_circ_0000512) was spliced from the RPPH1 gene (chr14:20811282-20811436) according to the annotation in circBase (http://www.circbase.org/). Its junction sites from back splicing were validated by Sanger sequencing (Fig. 1c, d).

We isolated six GSCs from clinical patients with WHO IV pathological diagnoses. The patient-derived glioma stem cells GSC35 and GSC38 were used to continue our study. We confirmed their stem-like properties by neurosphere formation and differentiation (Fig. S1b). Then, immunofluorescence staining was used to test the expression of stem cell markers (CD133 and Nestin) in the isolated neurospheres (Fig. S1a). In addition, differentiation markers GFAP and β-III tubulin were observed expressed in GSCs to validate their differentiation capacity (Fig. S1c). In this way, we identified GSC35 and GSC38 as glioma stem cells.

CircRPPH1 had a circular shape and could withstand RNase R digestion. So we performed RNase R qPCR and saw that circRPPH1 was expressed while the linear form of RPPH1 was digested (Fig. 1e). Northern blotting also revealed the presence of conjunction primers in cDNA while no products in gDNA (Fig. 1g). We then used FISH to show the position of circRPPH1 in the cytoplasm (Fig. 1f). Using clinical samples, we determined the circRPPH1 expression (70 patient-derived primary gliomas with 10 paired normal brain tissues) via qPCR. It showed that WHO IV GBM had the highest level of circRPPH1 expression compared to the others (Fig. 1h). Parents with high circRPPH1 expression had a shorter median survival time, according to Kaplan-Meier survival curves (Fig. 1i).

### Knockdown of circRPPH1 suppressed the malignant phenotype and stemness of GSCs in vitro

**Fig. 2:**
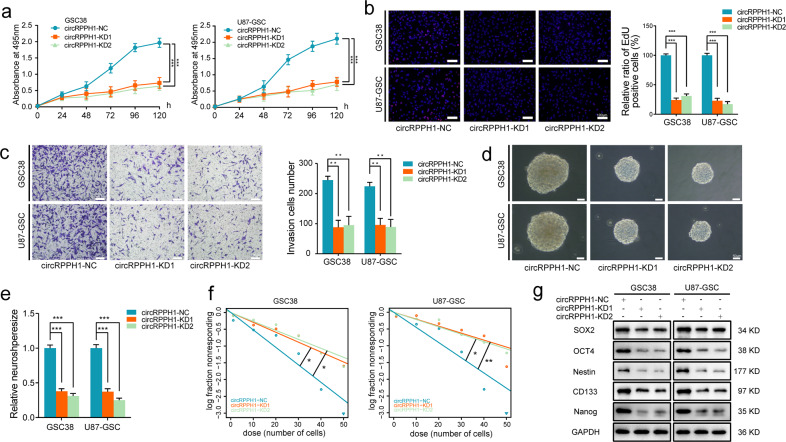
Knockdown of circRPPH1 suppressed the proliferation, invasion, self-renewal, and the expression of stemness in GSCs. **a** MTS assays showed that the cell viability of GSC38 and U87-GSC decreased after circRPPH1 knockdown. (GSC38, *p* < 0.001; U87-GSC, *p* < 0.001; Student’s t-test). **b** EdU assays showed the proliferation of GSC38 and U87-GSC decreased after circRPPH1 knockdown. Scale bar = 100 μm. (GSC38, *p* < 0.001; U87-GSC, *p* < 0.001; Student’s t-test). **c** Transwell assays showed the invasion abilities of GSC38 and U87-GSC were weakened under circRPPH1 knockdown. Scale bar = 100 μm. (GSC38, *p* < 0.01; U87-GSC, *p* < 0.01; Student’s t-test). **d**, **e** Neurospheres formation assays showed a decreased sphere size after circRPPH1 knockdown in GSC38 and U87-GSC. Scale bar = 50 μm. (GSC38, *p* < 0.001; U87-GSC, *p* < 0.001; Student’s t-test). **f** Extreme limiting dilution analysis showed the decreased stem cell enrichment under circRPPH1 knockdown. (GSC38, kd1: *p* = 0.0362, kd2: *p* = 0.0152; U87-GSC, kd1: *p* = 0.0066, kd2: *p* = 0.0104; ELDA analysis; circles represent corresponding points, triangles mean the point is outside of the log fraction number wells). **g** Stemness markers expression were found depleted in circRPPH1-knockdown GSC38 and U87-GSC by western blotting. NC: negative control, KD: knockdown. All results are obtained as the mean ± SD under at least biological triplicate assays. *p < 0.05, **p < 0.01, ***p < 0.001.

We designed two circRPPH1 junction-specific siRNA that could lower the amount of circRPPH1 in our GSCs. Overexpression was also used to boost the expression of circRPPH1. For circRPPH1 knockdown, we utilized GSC38 and U87-derived GSC, while for circRPPH1 overexpression, we chose GSC35 and LN229-derived GSC. Following that, we examined the expression of circRPPH1 after transfection (Fig. S2a, b). After that, MTS assays showed that knocking down circRPPH1 significantly reduced the viability of GSCs (Fig. 2a). The viability of GSCs, on the other hand, was considerably enhanced after circRPPH1 overexpression (Fig. S2c). EdU assays revealed that the circRPPH1 knockdown group contained only a few weak EdU-positive cells compared to the control group (Fig. 2b). Then, Transwell assays indicated that circRPPH1 knockdown significantly reduced the invasive ability of GSCs (Fig. 2c). Accordingly, when circRPPH1 was overexpressed, the number of EdU-positive cells increased (Fig. S2d). More invasive GSCs were also observed in transwell assays (Fig. S2e).

When knocking down circRPPH1, It showed that GSCs had a reduced ability to form spheres, and extreme limiting dilution assays revealed that the self-renewal ability of GSCs was significantly diminished (Fig. 2d–f). However, when overexpressing circRPPH1, neurosphere formation assays displayed a larger size of GSCs, and an enhanced self-renewal ability of GSCs was spotted by extreme limiting dilution assays (Fig. S2f, g). Finally, we used western blotting to test the expression of stemness markers in GSCs. It showed that circRPPH1 knockdown decreased the expression of stemness markers (SOX2, OCT4, Nestin, CD133, and Nanog) (Fig. 2g). In contrast, after overexpression of circRPPH1, the expression was increased (Fig. S2h).

### UPF1 can bind to and maintain the stability of circRPPH1, and circRPPH1 can mediate the promoting effects of UPF1 on GSCs

**Fig. 3:**
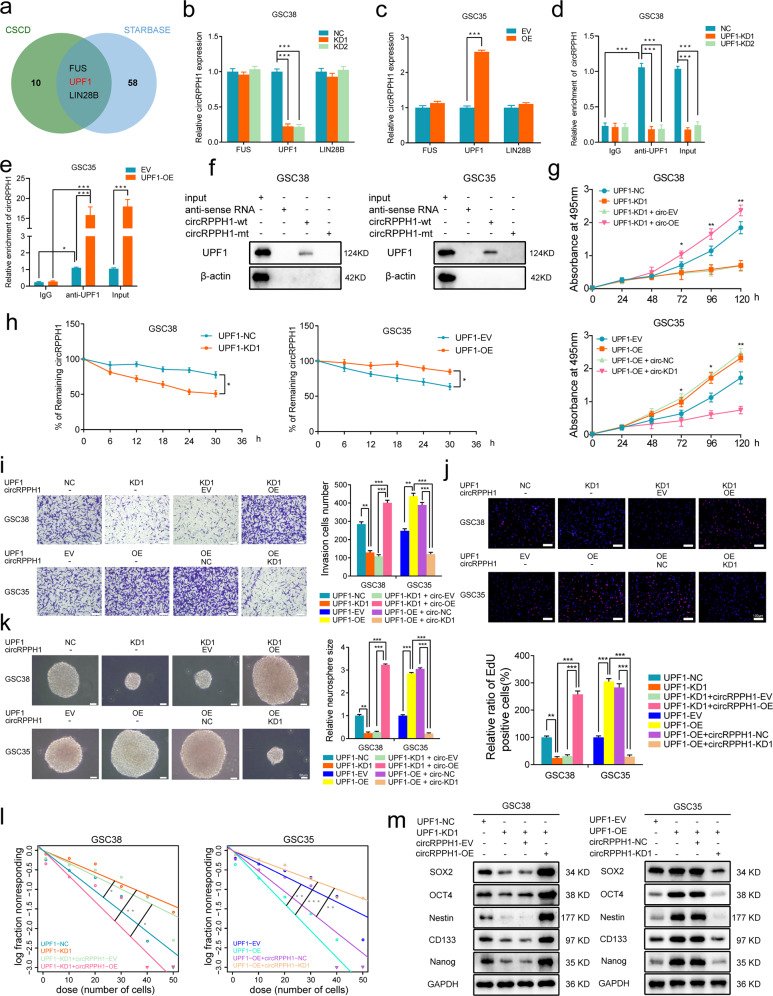
UPF1 binds to and maintains the stability of circRPPH1, and the binding mediates the promoting effects of UPF1 on GSCs. **a** Venn plot showed an intersection from two databases that predicted circRPPH1’s binding proteins. **b**, **c** Relative circRPPH1 expression of GSCs when knocking down or overexpressing proteins was tested via qPCR. (GSC38: *p* < 0.001; GSC35: *p* < 0.001; Student’s t-test). **d, e** RNA immunoprecipitation (RIP) assays were performed in GSC38 and GSC35 after UPF1 knockdown or overexpression, followed by qPCR to detect the enrichment of circRPPH1. (GSC38: *p* < 0.001; GSC35: *p* < 0.001; Student’s t-test). **f** RNA-pulldown followed by western blotting revealed the interaction between UPF1 and circRPPH1 in GSC38 and GSC35. **g** MTS assays showed that UPF1-KD reduced the viability of GSC38, which was reversed by circRPPH1-OE. UPF1-OE enhanced the viability of GSC35, which was rescued by circRPPH1-KD. (GSC38: *p* < 0.001; GSC35: *p* < 0.001; One-Way ANOVA). **h** Actinomycin D treatment revealed that UPF1-KD shortened the half-life of circRPPH1 while UPF1-OE prolonged it. (GSC38: *p* < 0.001; GSC35: *p* < 0.001; Student’s t-test). **i** Transwell assays showed that UPF1-KD reduced the invasion capacity of GSC38, which was rescued by circRPPH1-OE. UPF1-OE enhanced the invasion capacity of GSC35, which was rescued by circRPPH1-KD. Scale bar = 100 μm. (GSC38: *p* < 0.001; GSC35: *p* < 0.001; One-Way ANOVA). **j** EdU assays revealed that UPF1-KD reduced the proliferation of GSC38, which was rescued by circRPPH1-OE. UPF1-OE enhanced the proliferation of GSC35, which was rescued by circRPPH1-KD Scale bar = 100 μm. (GSC38: *p* < 0.001; GSC35: *p* < 0.001; One-Way ANOVA). **k** Neurosphere formation assays revealed that UPF1-KD reduced the sphere size of GSC38, which was rescued by circRPPH1-OE. UPF1-OE enhanced the sphere size of GSC35, which was rescued by circRPPH1-KD Scale bar = 50 μm. (GSC38: *p* < 0.001; GSC35: *p* < 0.001; One-Way ANOVA). **l** Extreme limiting dilution analysis showed that GSCs’ self-renewal was weakened by UPF1-KD, which could be reverted by circRPPH1-OE. UPF1-OE strengthened GSCs’ self-renewal, which was rescued by circRPPH1-KD. (GSC38, kd vs. nc: *p* = 0.0264, kd vs. kd+oe: *p* = 0.002; kd+ev vs. kd+oe: *p* = 0.0103; GSC35, oe vs. ev: *p* = 0.00615, oe vs. oe+ kd: *p* < 0.001, oe+nc vs. oe+kd: *p* = 0.00624; ELDA analysis; circles represent corresponding points, triangles mean the point is outside of the log fraction number wells). **m** Western blotting showed a significantly decreased stemness markers expression by UPF1-KD, which could be rescued by circRPPH1-OE. UPF1-OE could make an increased expression, which could be reverted by circRPPH1-KD. EV: empty vector, OE: overexpression, NC: negative control, KD: knockdown. All results are obtained as the mean ± SD under at least biological triplicate assays. *p < 0.05, **p < 0.01, ***p < 0.001.

To investigate the further mechanism that contributed to the high expression of circRPPH1, we explored the sequencing data in CSCD and Starbase databases. FUS, UPF1, and LIN28B were three proteins, as the intersection of two databases, that had the potential of binding circRPPH1 (Fig. 3a). We separately knocked down and overexpressed these proteins. Then through qPCR, it was discovered that only knocking down UPF1 reduced the level of circRPPH1. Reversely, the level increased when overexpressing UPF1. (Fig. 3b, c). Next, in GSCs, we applied RIP and RNA pull-down to validate the connection between circRPPH1 and UPF1 (Fig. 3d-f). Moreover, we found that UPF1 knockdown weakened the viability of GSC38 using MTS assays, which was rescued by overexpressing circRPPH1. In UPF1-overexpressed GSC35, cell viability was improved, and it was reverted by circRPPH1 knockdown (Fig. 3g). To further study the effect UPF1 brought to circRPPH1, we treated actinomycin D in UPF1-knockdown and UPF1-overexpression GSCs compared with their controls to block RNA producing. The remaining circRPPH1 expression showed a shortened half-life under UPF1 knockdown. If overexpressed UPF1, circRPPH1 existed longer. These findings suggested that UPF1 could help GSCs maintain stable circRPPH1 expression (Fig. 3h).

To ascertain the biological function of UPF1 in GSCs, we first conducted transwell and EdU assays. UPF1 knockdown weakened the invasive and proliferative capacities of GSC38, which was reversed by circRPPH1 overexpression. In contrast, when UPF1 got overexpressed, GSC35 exhibited an improved invasion and proliferation, which could be rescued through circRPPH1 knockdown (Fig. 3i, j). Neurosphere formation assays and extreme limiting dilution analysis were used to verify the stem-like properties of GSCs. They showed that UPF1 knockdown reduced the volume of neurospheres and inhibited GSC38’s self-renewal ability, which was rescued by circRPPH1-overexpression. UPF1 overexpression, on the other hand, improved GSC35’s stem-like properties to form neurospheres and self-renew. They were reversed with circRPPH1 knockdown (Fig. 3k, l). For the expression of stemness markers, we found them reduced when knocking down UPF1 and rescued with circRPPH1 overexpression in GSC38. While in UPF1-overexpressed GSC35, the opposite trends were observed (Fig. 3m).

### CircRPPH1 can regulate the protein stability and nuclear translocation of ATF3

**Fig. 4:**
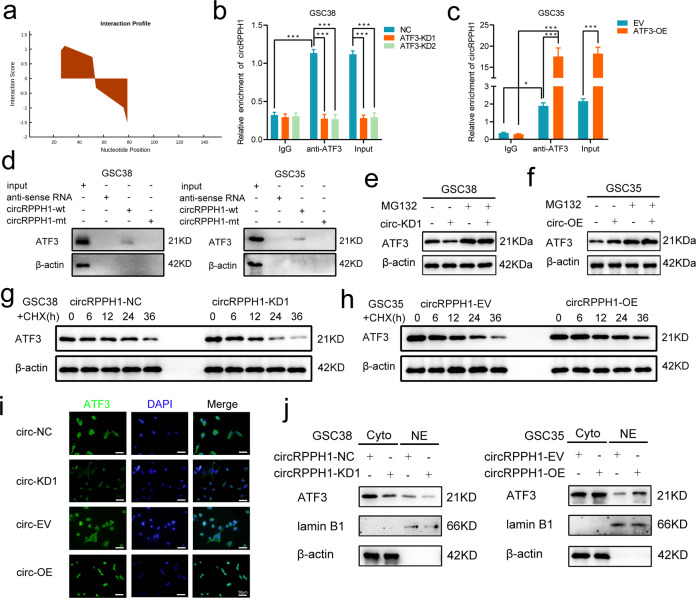
CircRPPH1 can regulate the stability and the nuclear translocation of ATF3. **a** The CatRAPID database predicted the interaction of circRPPH1 and ATF3. **b**, **c** RNA immunoprecipitation (RIP) assays were performed in GSCs after ATF3 knockdown and overexpression. The qPCR detected the expression of circRPPH1 in each group. (GSC38: *p* < 0.001; GSC35: *p* < 0.001; Student’s t-test). **d** RNA-pulldown and western blotting revealed the interaction between circRPPH1 and ATF3 in GSCs. **e**, **f** MG132 treated GSCs under ATF3 knockdown or overexpression was detected by western blotting using ATF3 antibodies. **g**, **h** Half-life of ATF3 was detected by western blotting after cycloheximide treatment for GSC38 (circRPPH1-KD) and GSC35 (circRPPH1-OE). **i** Immunofluorescence showed that circ-KD lowered ATF3 expression and that circ-OE resulted in the nuclear translocation of ATF3. Scale bar = 50 μm. **j** Western blotting showed that ATF3 expression was reduced by circRPPH1-KD. CircRPPH1-OE could make ATF3 undergo nuclear translocation. *EV* empty vector, *OE* overexpression, *NC* negative control, *KD* knockdown. All results are obtained as the mean ± SD under at least biological triplicate assays. *p < 0.05, **p < 0.01, ***p < 0.001.

To explore the role circRPPH1 plays downstream in GSCs, we predicted from the CatRAPID database and finally found that circRPPH1 could bind to ATF3 (Fig. 4a). We performed RIP experiments followed by qPCR. Knocking down ATF3 brought a decreased circRPPH1 enrichment in anti-ATF3 group compared with IgG group in GSC38 (Fig. 4b). Moreover, when ATF3 was overexpressed in GSC35, the expression of circRPPH1 increased accordingly (Fig. 4c). We subsequently used RNA pull-down with western blotting to confirm that circRPPH1 could interact with ATF3 in GSC38 and GSC35 (Fig. 4d).

To further investigate the effect of circRPPH1 on ATF3, we treated GSC38 and GSC35 with the proteasome inhibitor MG132. We found that ATF3 expression was decreased after circRPPH1 knockdown in GSC38 whereas it was increased with circRPPH1 overexpression inGSC35. With MG132 treatment, ATF3 displayed more expression which was almost unchanged following circRPPH1 alteration (Fig. 4e, f). So, we speculated that the presence of circRPPH1 may have protected ATF3 from degradation. Next, we treated GSCs with cycloheximide (CHX) to inhibit protein producing. Through western blot, we learned that circRPPH1 knockdown shortened the half-life of ATF3. With more circRPPH1, ATF3 could exist even longer (Fig. 4g, h). In other words, circRPPH1 guaranteed a more stable ATF3 expression.

We learned that ATF3 functioned as a transcription factor that could regulate gene transcription through nuclear translocation [36]. So immunofluorescence staining assays were used to locate ATF3. Results showed that ATF3 existed in the cytoplasm. CircRPPH1 knockdown decreased ATF3 expression in GSC38. When overexpressing circRPPH1 in GSC35, the binding effect made ATF3 translocate to the nucleus (Fig. 4i). To quantify the differences between the intranuclear and extranuclear expression of ATF3, we separately extracted the nuclear and the cytoplasmic lysates of GSCs. We used western blotting to find a weak intranuclear expression of ATF3 compared to the cytoplasm. When circRPPH1 was increased, intranuclear expression of ATF3 was elevated, which confirmed the nuclear translocation (Fig. 4j). However, the functions after that needed to be investigated.

### ATF3 knockdown can abolish circRPPH1 induced malignant phenotype of GSCs

**Fig. 5:**
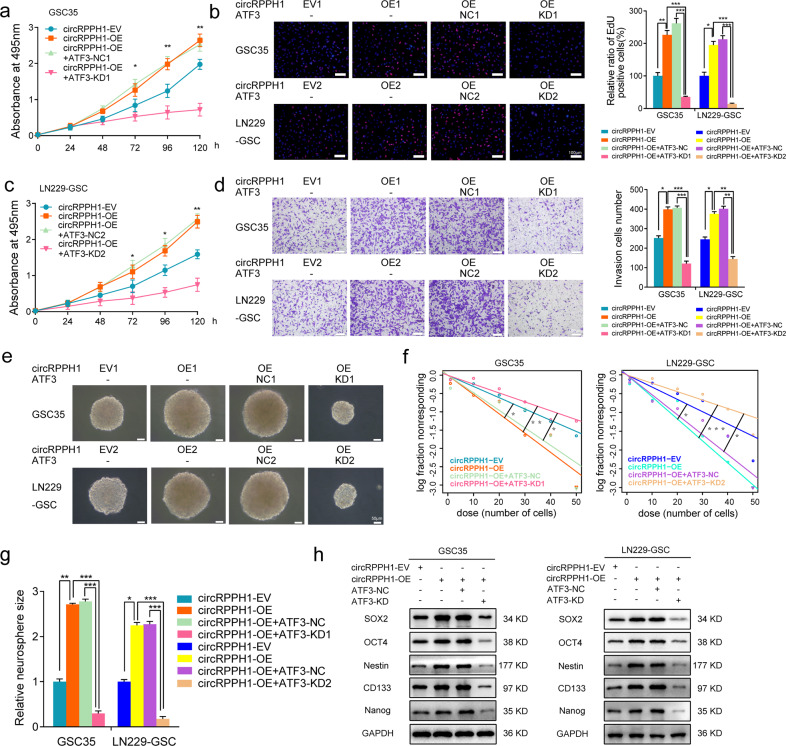
ATF3 knockdown can abolish the malignant phenotype of GSCs induced by circRPPH1. **a**, **c** MTS assays showed that the cell viability of GSCs with circRPPH1-OE was reduced by ATF3 knockdown. (GSC38: *p* < 0.01; LN229-GSC: *p* < 0.01; One-Way ANOVA). **b**, **d** EdU assays and Transwell assays showed that the proliferation and invasion abilities of circRPPH1-OE GSCs were weakened when knocking down ATF3. Scale bar = 100 μm. (GSC38: *p* < 0.01; LN229-GSC: *p* < 0.01; One-Way ANOVA). **e**–**g** Neurospheres formation assays and extreme limiting dilution analysis revealed the sphere-forming and self-renewing capacities of circRPPH1-OE GSCs were reduced under ATF3 knockdown. Scale bar = 50 μm. (GSC35, oe vs. ev: *p* = 0.0462, oe vs. oe+ kd: *p* = 0.00511, oe+nc vs. oe+kd: *p* = 0.014; LN229-GSC, oe vs. ev: *p* = 0.0302, oe vs. oe+ kd: *p* < 0.001, oe+nc vs. oe+kd: *p* = 0.00188; ELDA analysis; circles represent corresponding points, triangles mean the point is outside of the log fraction number wells). **h** Western blotting showed that the expression of stemness markers in circRPPH1-OE GSCs was deceased by ATF3 knockdown. All results are obtained as the mean ± SD under at least biological triplicate assays. *p < 0.05, **p < 0.01, ***p < 0.001.

Knowing that circRPPH1 can bind to ATF3, we knocked down ATF3 and used MTS assays to find that the cell viability caused by circRPPH1 overexpression was suppressed in GSC35 and LN229-GSC (Fig. 5a, c). EdU assay and transwell assays revealed that ATF3 knockdown could significantly inhibit the proliferation and invasive ability of GSCs gained by circRPPH1 overexpression (Fig. 5b, d). Neurosphere formation assays showed an attenuate neurosphere after ATF3 knockdown. It reverted the formation ability brought by circRPPH1 overexpression (Fig. 5e, g). Then, extreme limiting dilution analysis also showed a reduced self-renewal ability change in GSCs made by ATF3 knockdown (Fig. 5f). Finally, western blotting showed that ATF3 knockdown decreased the stemness expression of GSC35 and LN229-GSC. It successfully reversed the increased expression of stemness markers regulated by circRPPH1 overexpression (Fig. 5h).

### ATF3 can transcriptionally up-regulate UPF1 and nestin expression and maintain the stemness of GSCs

**Fig. 6:**
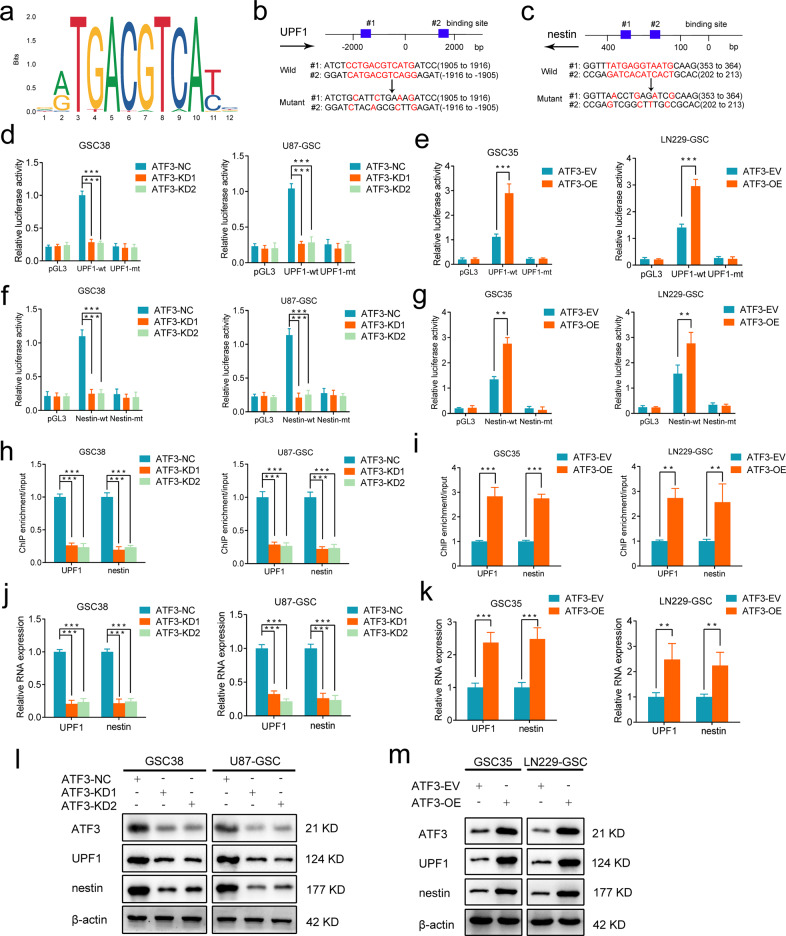
ATF3 can transcriptionally promote UPF1 and nestin expression and maintain the stemness of GSCs. **a** Sequence motif was calculated to show the consensus ATF3 binding motif through the JASPAR database. **b**, **c** Schematic diagram of ATF3 binding site in both of the 3′-UTR of UPF1 and Nestin. **d-g** The luciferase reporter gene assays showed that ATF3 knockdown (left) and overexpression (right) regulated the luciferase activities of UPF1 and Nestin. (GSC38, U87-GSC: *p* < 0.001; GSC35, LN229-GSC: *p* < 0.001; Student’s t-test). **h**, **i** ChIP qPCR assays showed a binding relationship between ATF3 and UPF1, Nestin. (GSC38, U87-GSC: *p* < 0.001; GSC35, LN229-GSC: *p* < 0.01; Student’s t-test). **j**, **k** QPCR showed that the RNA expression of UPF1 and Nestin was reduced by ATF3-KD and was increased by ATF3-OE. (GSC38, U87-GSC: *p* < 0.001; GSC35: *p* < 0.001; LN229-GSC: *p* < 0.01; Student’s *t*-test). **l**, **m** Western blotting revealed that ATF3-KD downregulated the expression of UPF1 and Nestin, while ATF3-OE upregulated the expression of UPF1 and Nestin. (GSC38, U87-GSC: *p* < 0.001; GSC35: *p* < 0.001; LN229-GSC: *p* < 0.01; Student’s t-test) EV: empty vector, OE: overexpression, NC: negative control, KD: knockdown. All results are obtained as the mean ± SD under at least biological triplicate assays. *p < 0.05, **p < 0.01, ***p < 0.001.

We then tried to explore ATF3’s role in the regulation of gene transcription. JASPAR database was applied to indicate that ATF3 could bind to UPF1 and Nestin’s promoter area (Fig. 6a). To investigate if ATF3 can regulate UPF1 and Nestin’s expression, we designed luciferase reporter genes with mutations in some loci of UPF1 and Nestin (Fig. 6b, c). We firstly knocked down the expression of ATF3. The luciferase reporter assays showed reduced luciferase activities of UPF1-wt and Nestin-wt in GSC38 and U87-GSC compared with the mutant groups (Fig. 6d, f). Then, the luciferase activities of UPF1-wt and Nestin-wt could be enhanced in ATF3-overexpressed GSC35 and LN229-GSC (Fig. 6e, g). Later, ChIP assays detected the degree of DNA binding. In GSC38 and U87-GSC, knocking down ATF3 resulted in a similar reduction in UPF1 and Nestin DNA expression. Overexpression of ATF3 could increase UPF1 and Nestin DNA enrichment in GSC35 and LN229-GSC (Fig. 6h, i). Finally, qPCR and western blotting experiments revealed a comparable trend in the RNA and protein expression of UPF1 and Nestin (Fig. 6j–m).

So far, we could conclude that ATF3 influenced the transcriptional levels of UPF1 and Nestin. UPF1/circRPPH1/ATF3 formed a positive feedback loop that supported GSCs’ malignant self-renewal.

### ATF3 can promote the malignant phenotype of GSCs via activating TGF-β1/Smad2 signaling

**Fig. 7:**
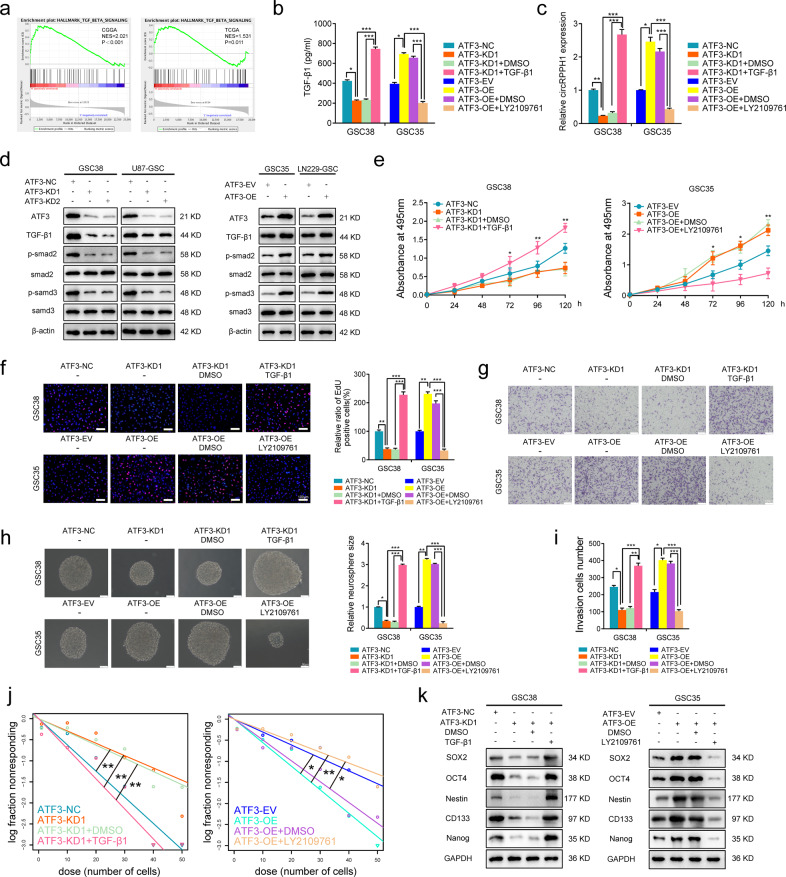
ATF3 can promote the malignant phenotype of GSCs via activating TGF-β1/Smad2 signaling. **a** Gene set enrichment analysis (GSEA) showed that high expression of ATF3 is positively associated with the TGF-β signaling pathway through data from the TCGA and CGGA datasets. (CGGA: *p* < 0.01; TCGA: *p* = 0.011). **b** ELISA assays detected the secretion of TGF-β1 after ATF3 knockdown or overexpression. (GSC38: *p* < 0.001; GSC35: *p* < 0.001; One-Way ANOVA). **c** The expression of circRPPH1 after ATF3 knockdown or overexpression with TGF-β1 or LY2109761 was detected by qPCR. (GSC38: *p* < 0.001; GSC35: *p* < 0.001; One-Way ANOVA). **d** Western blotting showed that ATF3-KD mediated the less expression of p-smad2 and p-smad3, while ATF3-OE elevated them. **e** MTS assays showed that the cell viability of ATF3-KD GSCs was improved by TGF-β1 treatment. And LY2109761 could inhibit the cell viability of ATF3-OE GSCs. (GSC38: *p* < 0.01; GSC35: *p* < 0.01; One-Way ANOVA). **f**, **g**, **i** EdU assays and Transwell assays showed the proliferation and invasion abilities of ATF3-KD GSCs were enhanced under TGF-β1 treatment. LY2109761 inhibited them in ATF3-OE GSCs Scale bar = 100 μm. (GSC38: *p* < 0.001; GSC35: *p* < 0.001; One-Way ANOVA). **h, j** Neurospheres formation assays and extreme limiting dilution analysis revealed that the sphere-forming and self-renewing capacity of ATF3-KD GSCs were enhanced under TGF-β1 treatment. LY2109761 could inhibit them in ATF3-OE GSCs Scale bar = 100 μm. (GSC38, kd vs. nc: *p* = 0.00621, kd vs. kd+TGFβ1: *p* = 0.0011; kd+DMSO vs. kd+ TGFβ1: *p* = 0.00228; GSC35, oe vs. ev: *p* = 0.0259, oe vs. oe+ LY2109761: *p* = 0.00534, oe+DMSO vs. oe+ LY2109761: *p* = 0.0184; ELDA analysis; circles represent corresponding points, triangles mean the point is outside of the log fraction number wells). **k** Western blotting showed that the stemness markers expression in ATF3-KD GSCs was upregulated by TGF-β1 treatment. And LY2109761 could inhibit the expression in ATF3-OE GSCs. All results are obtained as the mean ± SD under at least biological triplicate assays. *p < 0.05, **p < 0.01, ***p < 0.001.

We performed GSEA analysis using CGGA and TCGA databases to show the ATF3-mediated TGF-β pathway in GBM (Fig. 7a). According to ELISA assays, TGF-β1 release was dramatically inhibited when ATF3 was silenced in GSC38. In contrast, the TGF-β pathway inhibitor LY2109761 reduced the TGF-β1 release regulated by circRPPH1 overexpression (Fig. 7b). PCR showed that the addition of TGF-β1 could effectively elevate circRPPH1 expression in ATF3-knockdown GSC38. At the same time, LY2109761 decreased the level of circRPPH1 in ATF3-overexpressed GSC35 (Fig. 7c). Subsequently, western blotting showed that in GSC38 and U87-GSC, the expression of TGF-β1, p-smad2, and p-smad3 decreased together with ATF3-knockdown. In ATF3-overexpressed GSC35 and LN229-GSC, the expression of TGF-β1, p-smad2, and p-smad3 was upregulated (Fig. 7d).

Meanwhile, MTS assays, EdU proliferation assays, and transwell invasion assays told that in GSC38 TGF-β1 improved the viability, proliferative capacity, and invasive ability, which were inhibited by LY2109761 in ATF3-OE GSC35 (Fig. 7e–g). Finally, neurosphere formation assays, extreme limiting dilution analysis and related stemness markers expressions were also tested. All the above revealed that TGF-β1 could promote stem cell aggregation and self-renewal to form larger spheres and revert ATF3-knockdown mediated stemness markers expression (Fig. 7h–k).

### UPF1/ circRPPH1/ ATF3 feedback loop regulates glioma tumorigenesis in vivo

**Fig. 8:**
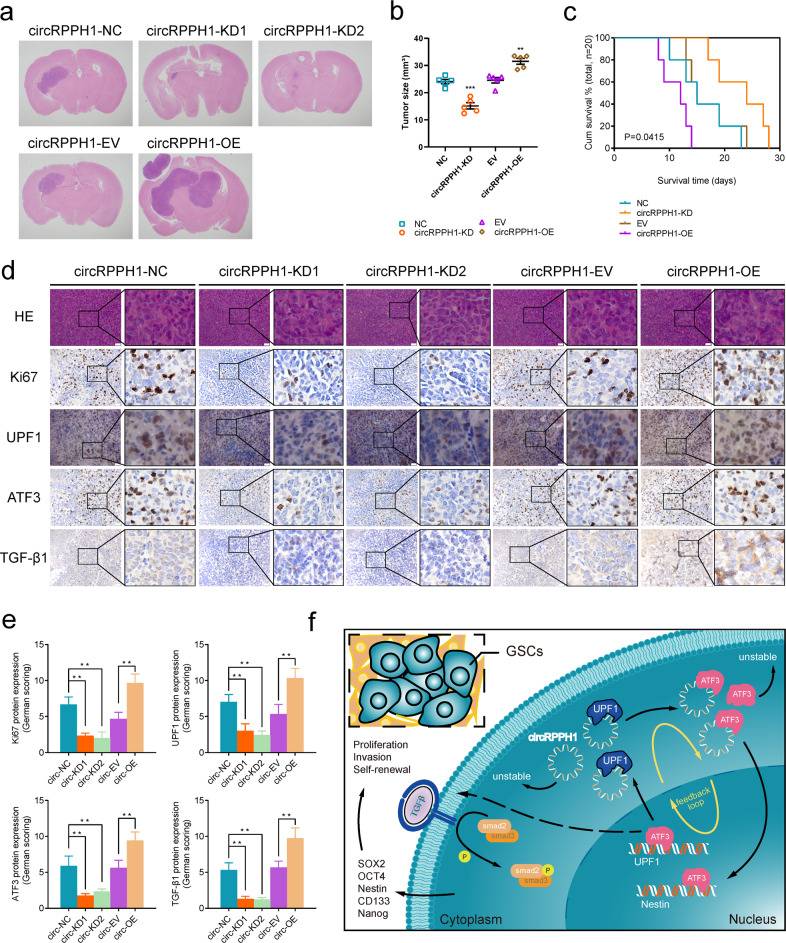
UPF1/circRPPH1/ATF3 feedback loop regulates glioma tumorigenesis in vivo. **a** Hematoxylin and eosin staining of intracranial tumor plantation showed the tumor size in the coronal location of eight groups. Scale bar = 10 mm. **b** The tumor volumes measured among eight groups are indicated. (nc vs. kd: *p* < 0.001; ev vs. oe: *p* = 0.0015; Student’s t-test). **c** Kaplan-Meier survival curves showed that circRPPH1-KD prolonged the survival of mice while circRPPH1-OE shortened the survival. (For each group, *n* = 5, nc vs. kd: *p* = 0.0415; ev vs. oe: *p* = 0.0180; Log-rank test). **d** Immunohistochemical staining showed that the protein expression was decreased by circRPPH1-KD and increased by circRPPH1-OE. Scale bar = 20 μm. **e** The German scoring of the protein expression in each group. (Ki67: *p* < 0.01; UPF1: *p* < 0.01; ATF3: *p* < 0.01; TGF-β1: *p* < 0.01 Student’s t-test). **f** Schematic diagram displayed the whole UPF1/circRPPH1/ATF3 feedback loop, which promoted the malignant phenotype of glioma and stemness of GSCs through the TGF-β1 signaling pathway. All results are obtained as the mean ± SD under at least biological triplicate assays. *p < 0.05, **p < 0.01, ***p < 0.001.

We also used intracranial carcinogenesis tests to confirm the impact of the UPF1/circRPPH1/ATF3 regulatory loop in vivo. CircRPPH1 knockdown can significantly reduce the size of brain tumors (Fig. 8a, b). The survival status of mice was also reported by Kaplan–Meier survival curves, which revealed that circRPPH1 knockdown prolonged the survival time of mice (Fig. 8c). Immunohistochemical staining (IHC) results showed that circRPPH1 knockdown led to lower levels of Ki67, UPF1, ATF3, and TGF-β1. And the increased expression of them was obtained by circRPPH1 overexpression (Fig. 8d). The protein expression was calculated using the German scoring method (Fig. 8d). Finally, we used a schematic diagram to illustrate the regulatory feedback loop (Fig. 8f). Our study, therefore, revealed an important feedback loop that explained the high malignancy of GBM.

## Discussion

Glioblastoma is a very aggressive and malignant disease with low survival rates and high recurrence rates. GSCs are a class of cells with self-renewing properties, which have a greater capacity to mediate their malignant proliferation, tissue invasion through their stemness [37, 38].

The role of circRNAs in tumors has been widely reported. Their unique stable structure, which often comes from RNA back-splicing, enables them to play a more persistent role in the molecular regulation process. Due to its resistance to RNase R digestion, we enriched it to investigate its biological function further. We examined the expression level and the localization of circRPPH1 using qPCR and FISH assays in GSCs. We then used clinical data and Kaplan-Meier survival analyses to find the correlation with poor overall survival. To confirm the biological function of circRPPH1, we performed siRNA knockdown. Then, we examined the changes in malignant behavior of GSCs using the MTS assays, EdU assays, and Transwell assays. They showed that circRPPH1 knockdown significantly reduced the malignant behavior.

CircRPPH1 has been mainly reported in breast cancer related to oncogenesis. These researches all take a start from circRPPH1 to study its downstream pathogenic mechanism, which invariably exerts regulatory effects by indirectly affecting other oncogenes through miRNAs sponging [39–43]. Another reported circRPPH1-related carcinoma in non-small cell lung cancer. Researchers validated the oncogene role of circRPPH1 and detected a role through PI3K/AKT and JAK2/STAT3 signaling axes and did not delve into the mechanisms involved [44].

Previous studies have revealed the role of circRNAs with miRNA sponging mechanisms in the development and treatment of GBM [45]. Recent studies have revealed that some circRNAs can translate a small peptide that plays a regulatory role in tumor development [46–49]. To explain the high expression of circRPPH1 within GBM, we tried to find evidence of interaction that is more direct and convincing and has clinical translational potential. We obtained three proteins that may have interactions and tested them with RIP assays and found that Upstream frameshift 1 (UPF1) strongly binds circRPPH1. UPF1 is an RNA helicase that regulates nonsense-mediated mRNA decay (NMD), which helps to target aberrant transcripts to protect cells from producing toxic protein [16, 17]. Finally, we found that UPF1 could keep circRPPH1 stable. In this way, circRPPH1 sustained and enhanced the malignant phenotype of GSCs slightly. So in gliomas, UPF1 could not only act with LncRNA PVT1 to aggravate the progression of glioma but can bind circRPPH1 to help strengthen the malignant phenotype [22].

To further reveal the downstream regulatory mechanism of circRPPH1, we continued to explore the CatRAPID database. It indicated that circRPPH1 bind to Activating transcription factor 3 (ATF3). ATF3 is a transcription factor with a unique leucine zipper (bZIP) domain structure. To clarify the specific role of the protein ATF3, we used CHX chasing to detect its half-life, and the results showed that circRPPH1 knockdown could reduce the half-life of ATF3. To investigate the localization of ATF3, we performed immunofluorescence experiments. When circRPPH1 was overexpressed, ATF3 protein underwent a significant nuclear translocation. Then, we wondered if it was related to the malignant behavior of GSC. So we proceeded to knock down ATF3 and demonstrated by the same phenotypic experiments that ATF3 can promote malignant progression of glioma.

Since ATF3 is a transcription factor, we used the database to predict its possible binding sequences. Interestingly, the results suggested that ATF3 might bind to UPF1 and Nestin sequences to promote its transcription. We verified this binding role using luciferase reporter assays. ATF3 could bind both UPF1 and Nestin’s sequences and promote transcription, which forms a positive feedback loop by activating UPF1 transcription. At the same time, Nestin is an important stemness marker and one of the sources of GSCs malignancy. To further identify the relevant signals, we performed a GSEA enrichment analysis, and the results suggested a close correlation with the TGF-β signaling pathway. We examined the relevant pathways, which showed that ATF3 exerts its regulatory role precisely through the TGF-β signaling pathway.

The stemness alteration of GSCs has been the focus of our study. The presence of tumor-initiating GSCs population attributed to the poor outcome of GBM, which has been shown to drive cell proliferation and invasion [50–52]. Our study identified a positive feedback UPF1/circRPPH1/ATF3 loop, and we examined the stemness changes in each regulatory section. At the same time, the regulatory loop we revealed happens to promote stemness regulation of Nestin. Therefore, we believe that such a conclusion can provide a new reference for revealing the mechanism of GSCs and developing anti-tumor therapy for GBM.

## Supplementary information

Supplementary figure and table legends

Figure S1

Figure S2

Figure S3

Supplementary Table 1

Supplementary Table 2

Supplementary Table 3

aj-checklist

nr-author-list-change-form

Western Blot Raw Data

## Data Availability

All the data obtained for the study could be available by inquiring the corresponding author.
