# Supplementary material for: UPF1/circRPPH1/ATF3 feedback loop promotes the malignant phenotype and stemness of GSCs
Source: Cell Death Dis. 2022 Jul 23;13(7):645. doi: 10.1038/s41419-022-05102-2 (PMC9308777; doi:10.1038/s41419-022-05102-2)
Supplement: Supplementary file 1 — Supplementary figure and table legends [file 41419_2022_5102_MOESM1_ESM.docx]

**Supplementary figure legends**

**Figure S1. Isolation and identification of patient-derived GSCs.**

**a** Immunofluorescence staining of CD133 and Nestin with IgG antibody as a negative control for the identification of the stemness markers. Scale bar = 50 μm

**b** Immunofluorescence staining of CD133 and Nestin in patient-derived GSC38 and GSC35. Scale bar = 50 μm.

**c** Neurospheres formation (upper panel) and differentiation (lower panel) of GSC38 and GSC35. Scale bar = 100 μm.

**d** Immunofluorescence staining showing differentiated GSCs expressing GFAP or β-III tubulin. Scale bar = 50 μm.

**Figure S2. Overexpression of circRPPH1 promoted the malignant phenotype and stemness of GSCs in vitro.**

**a** **b** The RNA expression of circRPPH1 after circRPPH1 knockdown (left) or overexpression (right), as detected by qPCR. (GSC38, U87-GSC: *p* ＜ 0.001; GSC35, LN229-GSC: *p* ＜ 0.001; Student’s t-test)

**c** MTS assays showed that the cell viability of GSC35 and LN229-GSC was enhanced by circRPPH1-OE. (GSC35: *p* ＜ 0.001; LN229-GSC: *p* ＜ 0.001; Student’s t-test)

**d** EdU assays showed that the proliferation of GSC35 and LN229-GSC was improved by circRPPH1-OE. Scale bar = 100 μm. (GSC35: *p* ＜ 0.01; LN229-GSC: *p* ＜ 0.001; Student’s t-test)

**e** Transwell assays showed that the invasion ability of GSC35 and LN229-GSC was improved by circRPPH1-OE. Scale bar = 100 μm. (GSC35: *p* ＜ 0.001; LN229-GSC: *p* ＜ 0.001; Student’s t-test)

**f** Neurospheres formation assays showed that the sphere size of GSC35 and LN229-GSC was increased by circRPPH1-OE. Scale bar = 50 μm. (GSC35: *p* ＜ 0.01; LN229-GSC: *p* = 0.0370; Student’s t-test)

**g** Extreme limiting dilution analysis showed an increased stem cell enrichment when circRPPH1 was overexpressing. (GSC35: *p* = 0.0219; LN229-GSC: *p* = 0.00449; ELDA analysis; circles represent corresponding points, triangles mean the point is outside of the log fraction number wells)

**h** Western blotting showed that the expression of stemness markers in GSC35 and LN229-GSC were upregulated by circRPPH1-OE. EV: empty vector, OE: overexpression, NC: negative control, KD: knockdown. All results are obtained as the mean ± SD under at least biological triplicate assays. * *p* < 0.05, ** *p* < 0.01, *** *p* < 0.001

**Figure S3. CircRPPH1 showed the highest expression among the top ten differentiated circRNAs in the GBM samples.**

For each group: *p* ＜ 0.001; Student’s t-test; All results are obtained as the mean ± SD under at least biological triplicate assays. * *p* < 0.05, ** *p* < 0.01, *** *p* < 0.001

**Supplementary table legends**

**Table S1. Clinical information on the isolated glioma stem cells (GSCs)**

**Table S2. siRNA sequences**

**Table S3. PCR Primers sequences**
