# Supplementary figures and images for: UPF1/circRPPH1/ATF3 feedback loop promotes the malignant phenotype and stemness of GSCs
Source: Cell Death Dis. 2022 Jul 23;13(7):645. doi: 10.1038/s41419-022-05102-2 (PMC9308777; doi:10.1038/s41419-022-05102-2)

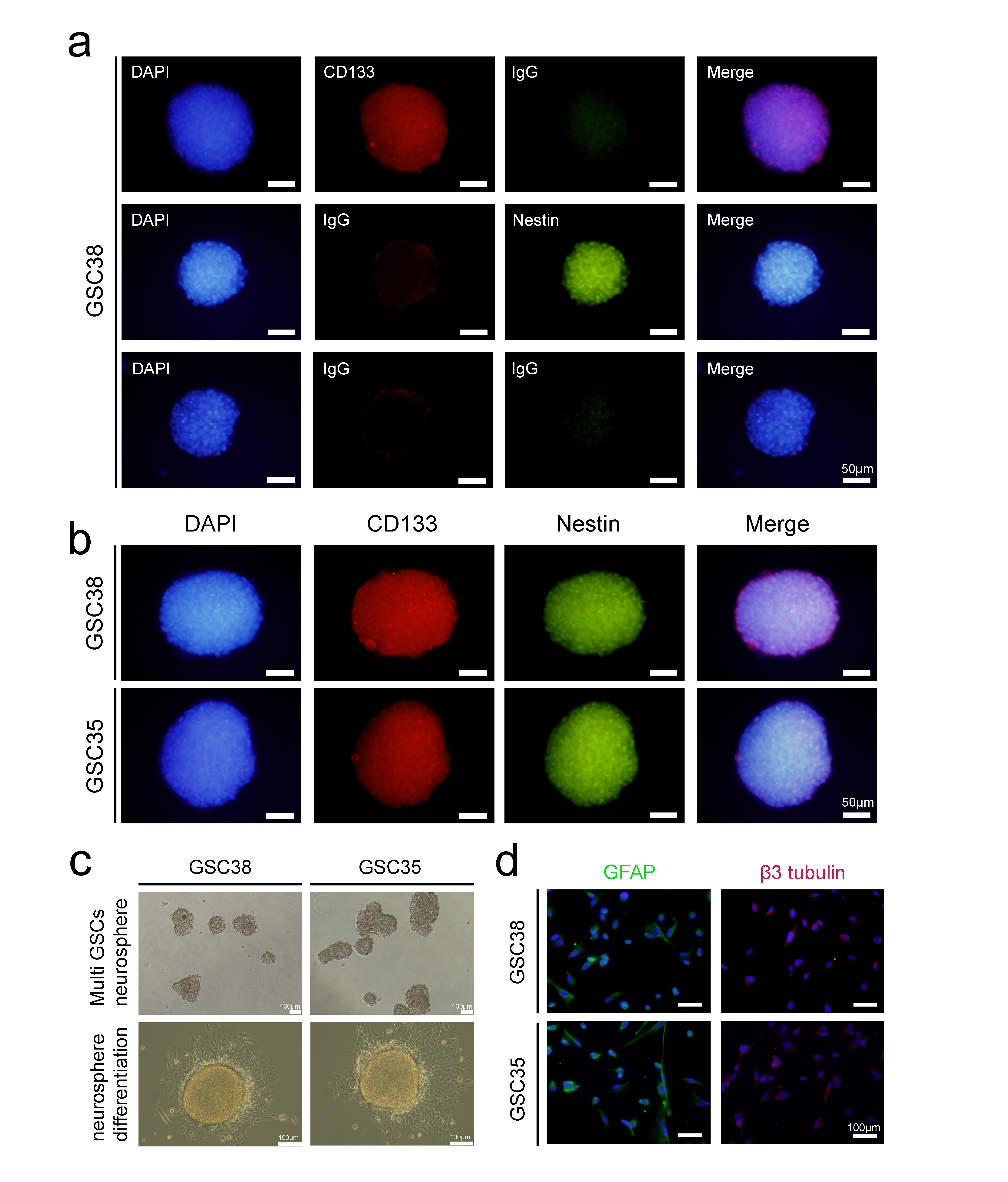

Supplement: Supplementary file 2 — Figure S1 [file 41419_2022_5102_MOESM2_ESM.png]

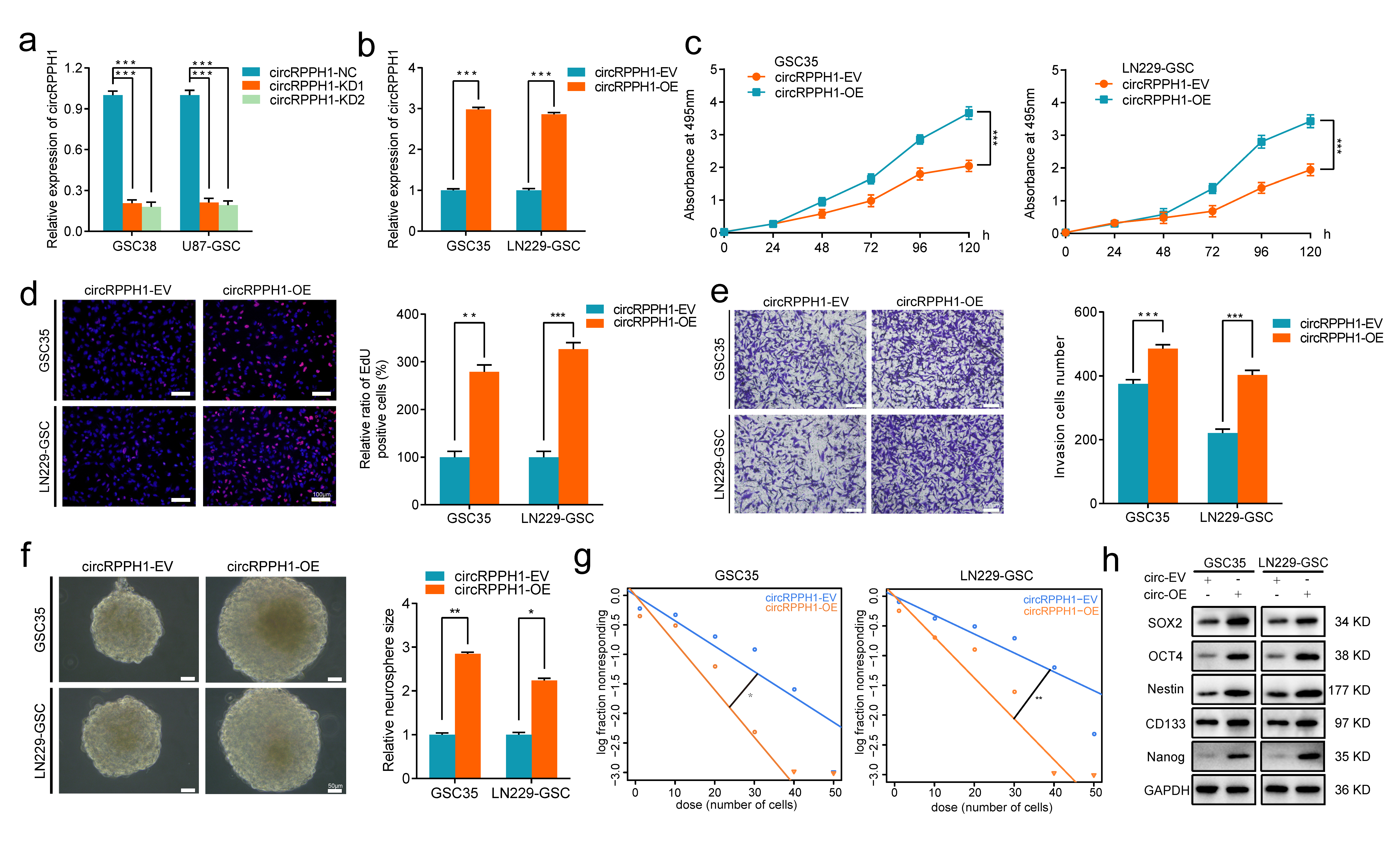

Supplement: Supplementary file 3 — Figure S2 [file 41419_2022_5102_MOESM3_ESM.png]

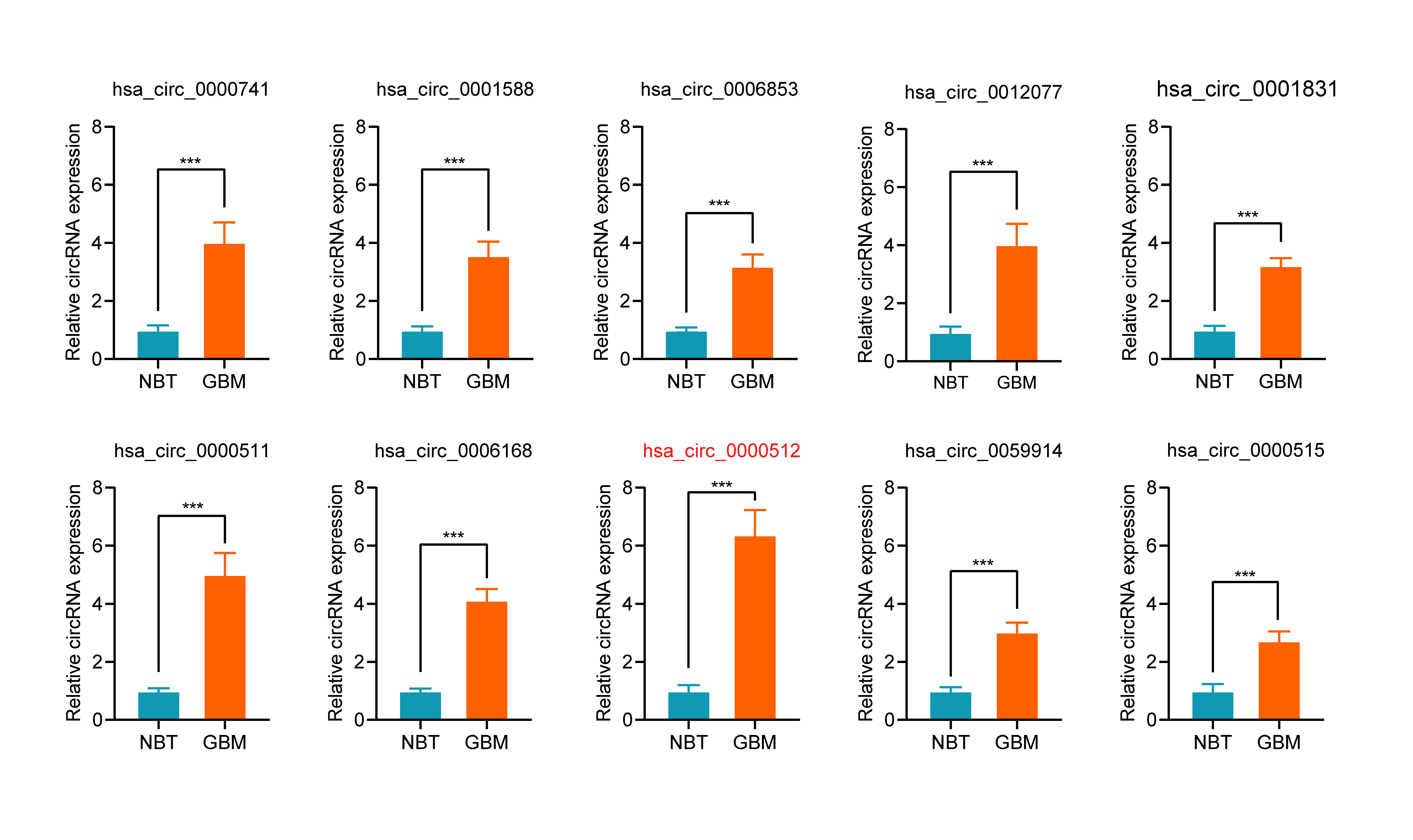

Supplement: Supplementary file 4 — Figure S3 [file 41419_2022_5102_MOESM4_ESM.png]
