# Supplementary material for: UPF1/circRPPH1/ATF3 feedback loop promotes the malignant phenotype and stemness of GSCs
Source: Cell Death Dis. 2022 Jul 23;13(7):645. doi: 10.1038/s41419-022-05102-2 (PMC9308777; doi:10.1038/s41419-022-05102-2)
Supplement: Supplementary file 5 — Supplementary Table 1 [file 41419_2022_5102_MOESM5_ESM.docx]

Supplementary Table 1. Clinical information on the isolated glioma stem cells (GSCs)

|  | GSC35 | GSC40 | GSC37 | GSC39 | GSC36 | GSC38 |
| --- | --- | --- | --- | --- | --- | --- |
| Gender | Male | Female | Male | Female | Female | Male |
| Age | 57 years old | 52 years old | 45 years old | 55 years old | 60 years old | 57 years old |
| Location | Right frontal lobe | Left insula | Left frontal lobe | Right parietal lobe | Left temporal lobe | Right occipital lobe |
| Pathological diagnosis | Glioblastoma | Glioblastoma | Glioblastoma | Glioblastoma | Glioblastoma | Glioblastoma |
| WHO grade | Ⅳ | Ⅳ | Ⅳ | Ⅳ | Ⅳ | Ⅳ |
| Ki-67 | 25% (+) | 30% (+) | 50% (+) | 55% (+) | 60% (+) | 60% (+) |
| IDH status | Wild | Wild | Wild | Wild | Wild | Wild |
| 1p/19q status | Non-codeletion | Non-codeletion | Non-codeletion | Non-codeletion | Non-codeletion | Non-codeletion |
| H3F3A status | Mutant | Mutant | Mutant | Mutant | Mutant | Mutant |
| MGMT status | Unmethylation | Unmethylation | Unmethylation | Unmethylation | Unmethylation | Unmethylation |
