# Supplementary material for: UPF1/circRPPH1/ATF3 feedback loop promotes the malignant phenotype and stemness of GSCs
Source: Cell Death Dis. 2022 Jul 23;13(7):645. doi: 10.1038/s41419-022-05102-2 (PMC9308777; doi:10.1038/s41419-022-05102-2)
Supplement: Supplementary file 6 — Supplementary Table 2 [file 41419_2022_5102_MOESM6_ESM.docx]

| **Gene** | **Forward sequences** | **Reverse sequences** |
| --- | --- | --- |
| circRPPH1-KD1 | GAGGUGAGUUCCCAGAGAA | UUCUCUGGGAACUCACCUC |
| circRPPH1-KD2 | GGAGCUUGGAACAGACUCA | UGAGUCUGUUCCAAGCUCC |
| UPF1-KD1 | UUCAUCUUCCUCGAAGUUCAA | UGUAAUAGGUGUCUUCUUCAU |
| UPF1-KD2 | GAAGAAGACACCUAUUACACG | GAAGAAGACACCUAUUACACG |
| ATF3-KD1 | GAGAAAUCCUCCUCUAUAUAG | GAGAAAUCCUCCUCUAUAUAG |
| ATF3-KD2 | AAAAUCCUUAGGAAACAGCAG | GCUGUUUCCUAAGGAUUUUCA |
| siRNA-NC | UUCUUCGAAGGUGUCACGUTT | ACGUGACACCUUCGAAGAATT |

**Supplementary Table 2. siRNA sequences**
