# Supplementary material for: UPF1/circRPPH1/ATF3 feedback loop promotes the malignant phenotype and stemness of GSCs
Source: Cell Death Dis. 2022 Jul 23;13(7):645. doi: 10.1038/s41419-022-05102-2 (PMC9308777; doi:10.1038/s41419-022-05102-2)
Supplement: Supplementary file 7 — Supplementary Table 3 [file 41419_2022_5102_MOESM7_ESM.docx]

**Supplementary Table 3. PCR Primers sequences**

**RT-qPCR Primers**

| **Gene** | **Forward Primer** | **Reverse Primer** |
| --- | --- | --- |
| circRPPH1 | TTTGCCGGAGCTTGGAACA | GGTCCACGGCATCTCCTG |
| UPF1 | ACCGACTTTACTCTTCCTAGCC | AGGTCCTTCGTGTAATAGGTGTC |
| ATF3 | CCTCTGCGCTGGAATCAGTC | TTCTTTCTCGTCGCCTCTTTTT |
| Nestin | CTGCTACCCTTGAGACACCTG | GGGCTCTGATCTCTGCATCTAC |
| GAPDH | GGAGCGAGATCCCTCCAAAAT | GGCTGTTGTCATACTTCTCATGG |
| β-actin | CATGTACGTTGCTATCCAGGC | CTCCTTAATGTCACGCACGAT |

**ChIP RT-qPCR Primers**

| **Gene** | **Forward Primer** | **Forward Primer** |
| --- | --- | --- |
| UPF1 | GCACAATCTTGGCTCACT | GCACAATCTTGGCTCACT |
| Nestin | GCAGGAGAATCACTTGAAC | CCACCAATATGTAGAATAGAGG |
