# Supplementary material for: UPF1/circRPPH1/ATF3 feedback loop promotes the malignant phenotype and stemness of GSCs
Source: Cell Death Dis. 2022 Jul 23;13(7):645. doi: 10.1038/s41419-022-05102-2 (PMC9308777; doi:10.1038/s41419-022-05102-2)
Supplement: Supplementary file 9 — nr-author-list-change-form [file 41419_2022_5102_MOESM9_ESM.pdf]

SPRINGER NATURE

In accordance with Springer Nature Authorship Policy we agree to change the authors of the manuscript as indicated below.

NAME OF JOURNAL: Cell Death & Disease

TITLE OF MANUSCRIPT: UPF1/circRPPH1/ATF3 feedback loop promotes the malignant phenotype and stemness of GSCs

MANUSCRIPT NUMBER: CDDIS-22-1409RR

CORRESPONDING AUTHORS NAME: Zhitao Jing, Yang Jiang

PREVIOUS AUTHOR NAMES:

Jinkun Xu, Yang Jiang, Guoqing Zhang, Jinpeng Hu, Hao Li, Junshuang Zhao, Shengliang Zong, Zhengting Guo, Zhitao Jing

UPDATED AUTHOR NAMES:

Jinkun Xu, Guoqing Zhang, Jinpeng Hu, Hao Li, Junshuang Zhao, Shengliang Zong, Zhengting Guo, Yang Jiang, Zhitao Jing

CHANGE TO AUTHOR LIST:

Jinkun Xu, Guoqing Zhang, Jinpeng Hu, Hao Li, Junshuang Zhao, Shengliang Zong, Zhengting Guo, Yang Jiang, Zhitao Jing

Print Name	Signature	Date
Jinkun Xu	Jinkun Xu	13th July, 2022
Guoqing Zhang	Guoqing Zhang	13th July, 2022
Jinpeng Hu	Jinpeng Hu	13th July, 2022
Hao Li	Hao Li	13th July, 2022
Junshuang Zhao	Junshuang Zhao	13th July, 2022
Shengliang Zong, Zhengting Guo	Shengliang Zong Zhengting Guo	13th July, 2022
Yang Jiang	Yang Jiang	13th July, 2022
Zhitao Jing	Zhitao Jing	13th July, 2022
