# Supplementary material for: UPF1/circRPPH1/ATF3 feedback loop promotes the malignant phenotype and stemness of GSCs
Source: Cell Death Dis. 2022 Jul 23;13(7):645. doi: 10.1038/s41419-022-05102-2 (PMC9308777; doi:10.1038/s41419-022-05102-2)
Supplement: Supplementary file 10 — Western Blot Raw Data [file 41419_2022_5102_MOESM10_ESM.docx]

**Western Blot Raw Data**

Figure 2

CircRPPH1-KD

















Figure S2

CircRPPH1-OE












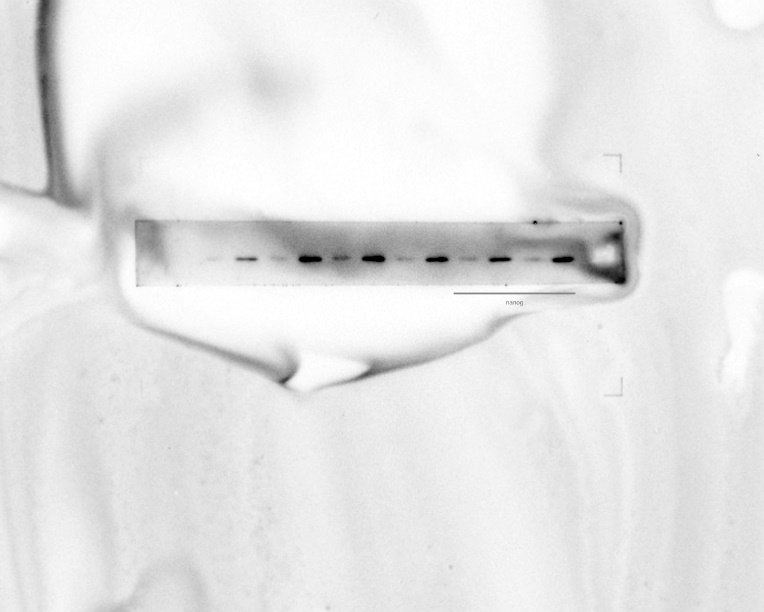




Figure 3

UPF1












Stemness marker

KD

















OE

















Figure 4

ATF3












MG132












CHX












NE & Cyto

















Figure 5

GSC35

















LN229

















Figure 6

KD












OE









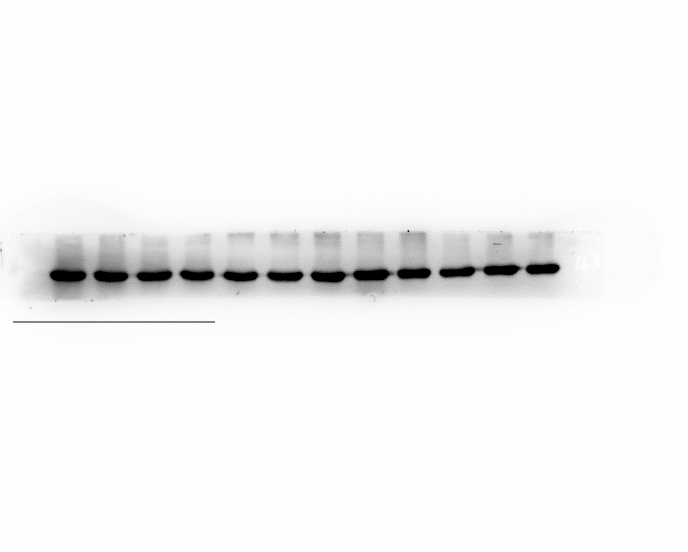


Figure 7

Signal pathway

KD















TGF-β







OE




















Stemness

KD

















OE
